# The genome sequence of the Yellow Meadow Ant, *Lasius flavus* (Fabricius, 1782) (Hymenoptera: Formicidae)

**DOI:** 10.12688/wellcomeopenres.27332.1

**Published:** 2026-08-26

**Authors:** Gavin R. Broad, Natural History Museum Genome Acquisition Lab, Darwin Tree of Life Barcoding Collective, Wellcome Sanger Institute Tree of Life Management, Samples and Laboratory team, Wellcome Sanger Institute Scientific Operations: Sequencing Operations, Wellcome Sanger Institute Tree of Life Core Informatics team, Tree of Life Core Informatics collective, Darwin Tree of Life Consortium

**Affiliations:** 1 Natural History Museum, London, England, UK

**Keywords:** Lasius flavus, Yellow Meadow Ant, genome sequence, chromosomal, Hymenoptera

## Abstract

We present a genome assembly from an individual female *Lasius flavus* (Yellow Meadow Ant; Arthropoda; Insecta; Hymenoptera; Formicidae). The assembly contains two haplotypes with total lengths of 314.61 megabases and 325.88 megabases. Most of haplotype 1 (95.46%) and haplotype 2 (92.16%) are each scaffolded into 15 chromosomal pseudomolecules. The mitochondrial genome has also been assembled, with a length of 21.36 kilobases. This assembly was generated as part of the Darwin Tree of Life project, which produces genomes for eukaryotic species found in Britain and Ireland.

## Species taxonomy

Eukaryota; Opisthokonta; Metazoa; Eumetazoa; Bilateria; Protostomia; Ecdysozoa; Panarthropoda; Arthropoda; Mandibulata; Pancrustacea; Altocrustacea; Allotriocarida; Hexapoda; Insecta; Dicondylia; Pterygota; Neoptera; Eumetabola; Endopterygota; Hymenoptera; Apocrita; Aculeata; Formicoidea; Formicidae; Formicinae; Lasiini; *Lasius*; *Cautolasius*; *Lasius flavus* (Fabricius, 1782) (NCBI:txid67763)

## Background

The nest mounds of *Lasius flavus*, the Yellow Meadow Ant ( Figure 1), are a familiar sight in European grassland. Where the ant is common, these mounds impart a bumpy appearance to the ground, with nests persisting for decades. Although best known as a mound-building meadow ant in Britain, *L. flavus* inhabits a range of habitats and frequently founds nests under stones. *Lasius flavus* can be identified relatively easily by the uniformly yellow colour of the workers and long setae on the metasoma, amongst other features. It was thought that *L. flavus* occurs in North America too, but that population has been recognised as a separate species, *Lasius brevicornis* Emery ( Schär *et al.*, 2018).

**Figure 1.**
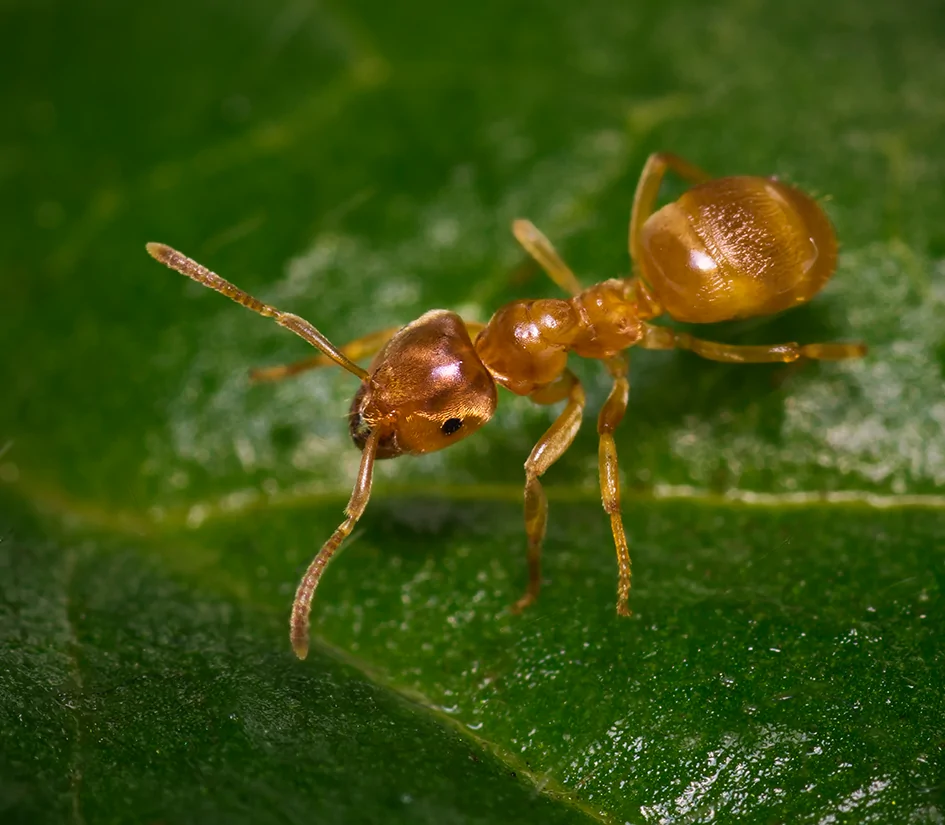
Lasius flavus. Photo credit: AJC1 via Wikimedia Commons.

King (2025) gives a detailed account of the role of *L. flavus* in creating and maintaining distinct grassland communities. The soil structure is changed by the ants, patches of bare earth are created, and the mounds offer different heights and aspects within a meadow, providing areas for Orthoptera to lay eggs, and places for winter annuals and certain mosses to grow. The ants are also food for various species, most notably Green Woodpeckers ( *Picus viridis*). Workers of *L. flavus* are hypogaeic, thought to eat mainly honeydew from root-feeding aphids and some small insects. Over the winter, the ants store aphids and eggs in parts of their nest and introduce them to roots in the spring.

While *L. flavus* is a common lowland species in the northern and temperate parts of its range, in more southern areas it is a species of mountains and upland forests ( Borowiec & Salata, 2022). In Britain, *L. flavus* is very widespread and one of our most abundant ants, although more local in the North of Scotland ( Fox, 2012). King (2025) reports a density of 17 255 worker yellow ants per m ^2^ in Richmond Park, London. Alates fly in the summer, most frequently in August and early September, with mated queens often crowding together in holes and under stones, frequently founding nests as groups ( Bolton & Collingwood, 1975), although in mature nests there are usually only one or two queens ( Boomsma *et al.*, 1993).

This chromosomal genome adds to a growing body of ant genomes which is opening up new fields of research into the genomes of these eusocial ecosystem engineers (e.g., Vizueta *et al.*, 2025). Other assemblies for this species are also available, including chromosome-level assemblies (GCA_048181785.1, GCA_048543215.1) (data obtained via NCBI Datasets, O’Leary *et al.*, 2024).

## Methods

### Sample acquisition and DNA barcoding

The specimen used for genome sequencing was an adult female *Lasius flavus* (specimen ID NHMUK010635107, ToLID iyLasFlav2). The same specimen was also used for Hi-C sequencing. It was collected from Tonbridge, England, UK (latitude 51.193, longitude 0.2742) on 2020-08-20 by Gavin Broad, who also identified it.

The initial identification was verified by an additional DNA barcoding process according to the framework developed by Twyford *et al.* (2024). A small sample was dissected from the specimen and stored in ethanol, while the remaining parts were shipped on dry ice to the Wellcome Sanger Institute (WSI) (see the protocol). The tissue was lysed, the COI marker region was amplified by PCR, and amplicons were sequenced and compared to the BOLD database, confirming the species identification ( Crowley *et al.*, 2023). Following whole genome sequence generation, the relevant DNA barcode region was also used alongside the initial barcoding data for sample tracking at the WSI ( Twyford *et al.*, 2024). The standard operating procedures for Darwin Tree of Life barcoding are available on protocols.io.

### Nucleic acid extraction

Detailed protocols for nucleic acid extraction developed at the Wellcome Sanger Institute (WSI) Tree of Life Core Laboratory are available on protocols.io ( Howard *et al.*, 2025). The iyLasFlav2 specimen was weighed and triaged to determine the appropriate extraction protocol. For high molecular weight (HMW) DNA extraction, 13.0 mg of head and thorax tissue was used. Tissue from the head and thorax was homogenised by powermashing using a PowerMasher II tissue disruptor. HMW DNA was extracted in the WSI Scientific Operations core using the Automated MagAttract v2 protocol. We used centrifuge-mediated fragmentation to produce DNA fragments in the 8–10 kb range, following the Covaris g-TUBE protocol for ultra-low input (ULI). Sheared DNA was purified by manual SPRI (solid-phase reversible immobilisation). The concentration of the sheared and purified DNA was assessed using a Nanodrop spectrophotometer and Qubit Fluorometer using the Qubit dsDNA High Sensitivity Assay kit. Fragment size distribution was evaluated by running the sample on the FemtoPulse system. For this sample, the final post-shearing DNA had a Qubit concentration of 0.99 ng/μL and a yield of 57.42 ng, with a fragment size of 13.8 kb. The 260/280 spectrophotometric ratio was 0.47, and the 260/230 ratio was 0.21. The Genomic Quality Number (GQN) was 7.1.

### PacBio HiFi library preparation and sequencing

Library preparation and sequencing were performed at the WSI Scientific Operations core. Prior to library preparation, the DNA was fragmented to ~10 kb. Ultra-low-input (ULI) libraries were prepared using the PacBio SMRTbell ^®^ Express Template Prep Kit 2.0 and gDNA Sample Amplification Kit. Samples were normalised to 20 ng DNA. Single-strand overhang removal, DNA damage repair, and end-repair/A-tailing were performed according to the manufacturer’s instructions, followed by adapter ligation. A 0.85× pre-PCR clean-up was carried out with Promega ProNex beads.

The DNA was evenly divided into two aliquots for dual PCR (reactions A and B), both following the manufacturer’s protocol. A 0.85× post-PCR clean-up was performed with ProNex beads. DNA concentration was measured using a Qubit Fluorometer v4.0 (Thermo Fisher Scientific) with the Qubit HS Assay Kit, and fragment size was assessed on an Agilent Femto Pulse Automated Pulsed Field CE Instrument (Agilent Technologies) using the gDNA 55 kb BAC analysis kit. PCR reactions A and B were then pooled, ensuring a total mass of ≥500 ng in 47.4 μl.

The pooled sample underwent another round of DNA damage repair, end-repair/A-tailing, and hairpin adapter ligation. A 1× clean-up was performed with ProNex beads, followed by DNA quantification using the Qubit and fragment size analysis using the Agilent Femto Pulse. Size selection was performed on the Sage Sciences PippinHT system, with target fragment size determined by Femto Pulse analysis (typically 4–9 kb). Size-selected libraries were cleaned with 1.0× ProNex beads and normalised to 2 nM before sequencing.

The sample was sequenced on a Revio instrument (Pacific Biosciences, California, USA). Prepared libraries with a molarity greater than 2 nM were normalised to 2 nM, and 15 μL was used for making complexes. For libraries with a molarity below 2 nM, the available 10 μL library volume was used without normalisation. Primers were annealed and polymerases were bound to generate circularised complexes, following the manufacturer’s instructions. Complexes were purified using 1.2× SMRTbell beads, diluted to the Revio loading concentration of 200–300 pM, and spiked with a Revio sequencing internal control. The sample was sequenced on a Revio 25M SMRT cell. SMRT Link software (Pacific Biosciences), a web-based workflow manager, was used to configure and monitor the run and to carry out primary and secondary data analysis.

### Hi-C

***Sample preparation and crosslinking***

The Hi-C sample was prepared from 20–50 mg of frozen head and thorax tissue from the iyLasFlav2 sample using the Arima-HiC v2 kit (Arima Genomics). Following the manufacturer’s instructions, tissue was fixed and DNA crosslinked using TC buffer to a final formaldehyde concentration of 2%. The tissue was homogenised using the Diagnocine Power Masher-II. Crosslinked DNA was digested with a restriction enzyme master mix, biotinylated, and ligated. Clean-up was performed with SPRISelect beads before library preparation. DNA concentration was measured with the Qubit Fluorometer (Thermo Fisher Scientific) and Qubit HS Assay Kit. The biotinylation percentage was estimated using the Arima-HiC v2 QC beads.

***Hi-C library preparation and sequencing***

Biotinylated DNA constructs were fragmented using a Covaris E220 sonicator and size selected to 400–600 bp using SPRISelect beads. DNA was enriched with Arima-HiC v2 kit Enrichment beads. End repair, A-tailing, and adapter ligation were carried out with the NEBNext Ultra II DNA Library Prep Kit (New England Biolabs), following a modified protocol where library preparation occurs while DNA remains bound to the Enrichment beads. Library amplification was performed using KAPA HiFi HotStart mix and a custom Unique Dual Index (UDI) barcode set (Integrated DNA Technologies). Depending on sample concentration and biotinylation percentage determined at the crosslinking stage, libraries were amplified with 10–16 PCR cycles. Post-PCR clean-up was performed with SPRISelect beads. Libraries were quantified using the AccuClear Ultra High Sensitivity dsDNA Standards Assay Kit (Biotium) and a FLUOstar Omega plate reader (BMG Labtech).

Prior to sequencing, libraries were normalised to 10 ng/μL. Normalised libraries were quantified again to create equimolar and/or weighted 2.8 nM pools. Pool concentrations were checked using the Agilent 4200 TapeStation (Agilent) with High Sensitivity D500 reagents before sequencing. Sequencing was performed using paired-end 150 bp reads on the Illumina NovaSeq 6000.

### Genome assembly

Prior to assembly of the PacBio HiFi reads, a database of *k*-mer counts ( *k* = 31) was generated from the filtered reads using FastK. GenomeScope2 ( Ranallo-Benavidez *et al.*, 2020) was used to analyse the *k*-mer frequency distributions, providing estimates of genome size, heterozygosity, and repeat content.

The HiFi reads were assembled using Hifiasm in Hi-C phasing mode ( Cheng *et al.*, 2021, 2022), producing two haplotypes. Hi-C reads ( Rao *et al.*, 2014) were mapped to the primary contigs using bwa-mem2 ( Vasimuddin *et al.*, 2019). Contigs were further scaffolded with Hi-C data in YaHS ( Zhou *et al.*, 2023), using the --break option for handling potential misassemblies. The scaffolded assemblies were evaluated using Gfastats ( Formenti *et al.*, 2022), BUSCO ( Manni *et al.*, 2021) and MerquryFK ( Rhie *et al.*, 2020).

The mitochondrial genome was assembled using MitoHiFi ( Uliano-Silva *et al.*, 2023). The MitoHiFi reference was the mitochondrial genome of *Lasius spathepus* (NC_053901.1).

### Assembly curation

The assembly was decontaminated using the Assembly Screen for Cobionts and Contaminants ( ASCC) pipeline. TreeVal was used to generate the flat files and maps for use in curation. Manual curation was conducted primarily in PretextView and HiGlass ( Kerpedjiev *et al.*, 2018). Scaffolds were visually inspected and corrected as described by Howe *et al*. (2021). Manual corrections included 254 breaks and 533 joins. This reduced the scaffold count by 42.8%, increased the scaffold N50 by 9.7%, and reduced the total assembly length by 5.0%. The curation process is described at sanger-tol/curation-resources . PretextSnapshot was used to generate a Hi-C contact map of the final assembly.

### Assembly quality assessment

The MerquryFK tool ( Rhie *et al.*, 2020) was run in a Singularity container ( Kurtzer *et al.*, 2017) to evaluate *k*-mer completeness and assembly quality for both haplotypes using the *k*-mer database ( *k* = 31) computed prior to genome assembly. The analysis outputs included assembly QV scores and completeness statistics.

The genome was analysed using the BlobToolKit pipeline, a Nextflow implementation of the earlier Snakemake version ( Challis *et al.*, 2020). PacBio reads were aligned to the assembly using minimap2 ( Li, 2018) and processed with SAMtools ( Danecek *et al.*, 2021) to generate coverage tracks. The pipeline runs BUSCO ( Manni *et al.*, 2021) using lineages identified from the NCBI Taxonomy ( Schoch *et al.*, 2020). For the three basal BUSCO lineages, eukaryota, bacteria and archaea, BUSCO genes are aligned to the UniProt Reference Proteomes database ( Bateman *et al.*, 2023) using DIAMOND BLASTp ( Buchfink *et al.*, 2021). The genome assembly is divided into chunks based on the density of BUSCO genes from the closest taxonomic lineage, and each chunk is aligned to the UniProt Reference Proteomes database using DIAMOND BLASTx. Sequences without DIAMOND BLASTx hits are extracted using seqtk and aligned to the NT database using BLASTn ( Altschul *et al.*, 1990). The outputs are consolidated into a BlobDir for visualisation in BlobToolKit. The BlobToolKit pipeline was developed using nf-core tooling ( Ewels *et al.*, 2020) and MultiQC ( Ewels *et al.*, 2016), with containerisation through Docker ( Merkel, 2014) and Singularity ( Kurtzer *et al.*, 2017).

## Genome sequence report

### Sequence data

PacBio sequencing of the *Lasius flavus* specimen generated 84.09 Gb (gigabases) from 7.44 million reads, which were used to assemble the genome. GenomeScope2.0 analysis using a *p* = 2 model gave a *k*-mer-based genome size estimate of 265.81 Mb, with a heterozygosity of 0.51% and repeat content of 20.71% ( Figure 2). These estimates guided expectations for the assembly. Based on the estimated genome size, the sequencing data provided approximately 267× coverage. Hi-C sequencing produced 112.13 Gb from 371.29 million reads, which were used to scaffold the assembly. Table 1 summarises the specimen and sequencing details.

**Figure 2.**
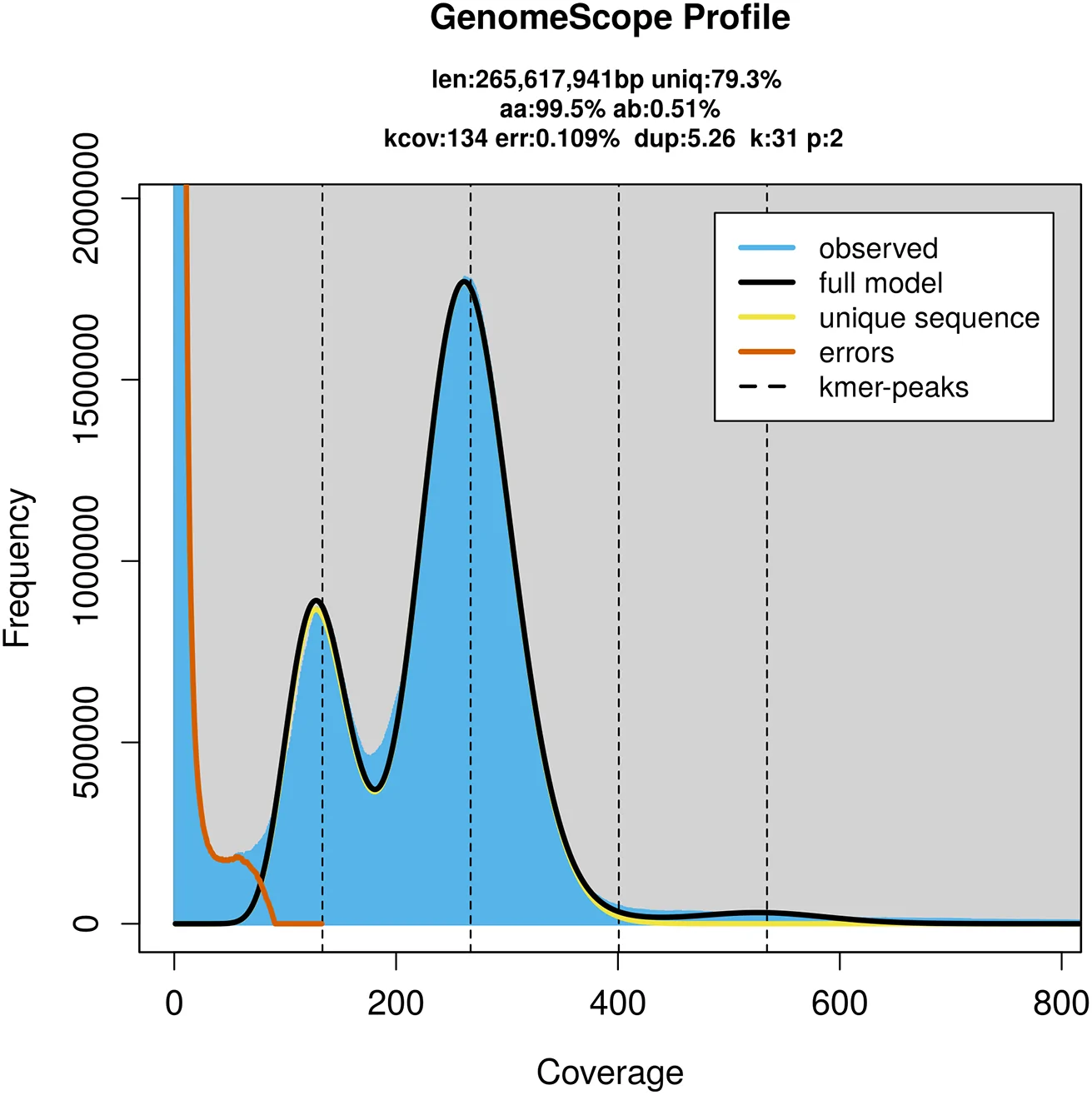
Frequency distribution of *k*-mers generated using GenomeScope2. The plot shows observed and modelled *k*-mer spectra, providing estimates of genome size, heterozygosity, and repeat content based on unassembled sequencing reads.

**Table 1.** Specimen and sequencing data for *Lasius flavus* (BioProject PRJEB88760).

Platform	PacBio HiFi	Hi-C
**ToLID**	iyLasFlav2	iyLasFlav2
**Specimen ID**	NHMUK010635107	NHMUK010635107
**BioSample (source individual)**	SAMEA8534278	SAMEA8534278
**BioSample (tissue)**	SAMEA8534281	SAMEA8534281
**Tissue**	head and thorax	head and thorax
**Instrument**	Revio	Illumina NovaSeq 6000
**Run accessions**	ERR14886544	ERR14895955
**Read count total**	7.44 million reads	371.29 million read pairs
**Base count total**	84.09 Gb	112.13 Gb

### Assembly statistics

The genome was assembled into two haplotypes using Hi-C phasing. Haplotype 1 was curated to chromosome level, while haplotype 2 was assembled to scaffold level. The final assembly has a total length of 314.61 Mb in 665 scaffolds, with 761 gaps, and a scaffold N50 of 20.88 Mb ( Table 2).

**Table 2.** Genome assembly statistics for *Lasius flavus.*

Genome assembly	Haplotype 1	Haplotype 2
**Assembly name**	iyLasFlav2.hap1.1	iyLasFlav2.hap2.1
**Assembly accession**	GCA_965645265.1	GCA_965645255.1
**Assembly level**	chromosome	chromosome
**Span (Mb)**	314.61	325.88
**Number of chromosomes**	15	15
**Number of contigs**	1 426	1 290
**Contig N50**	0.81 Mb	0.87 Mb
**Number of scaffolds**	665	617
**Scaffold N50**	20.88 Mb	20.78 Mb
**Longest scaffold length (Mb)**	26.26	26.73
**Organelles**	Mitochondrial genome: 21.36 kb	-

Most of the haplotype 1 assembly sequence (95.46%) was assigned to 15 chromosomal-level scaffolds. These chromosome-level scaffolds, confirmed by Hi-C data, are named according to size ( Figure 3; Table 3). The exact order and orientation of the contigs on chromosome 4 (21 007–22 100 Kbp) are unknown.

**Figure 3.**
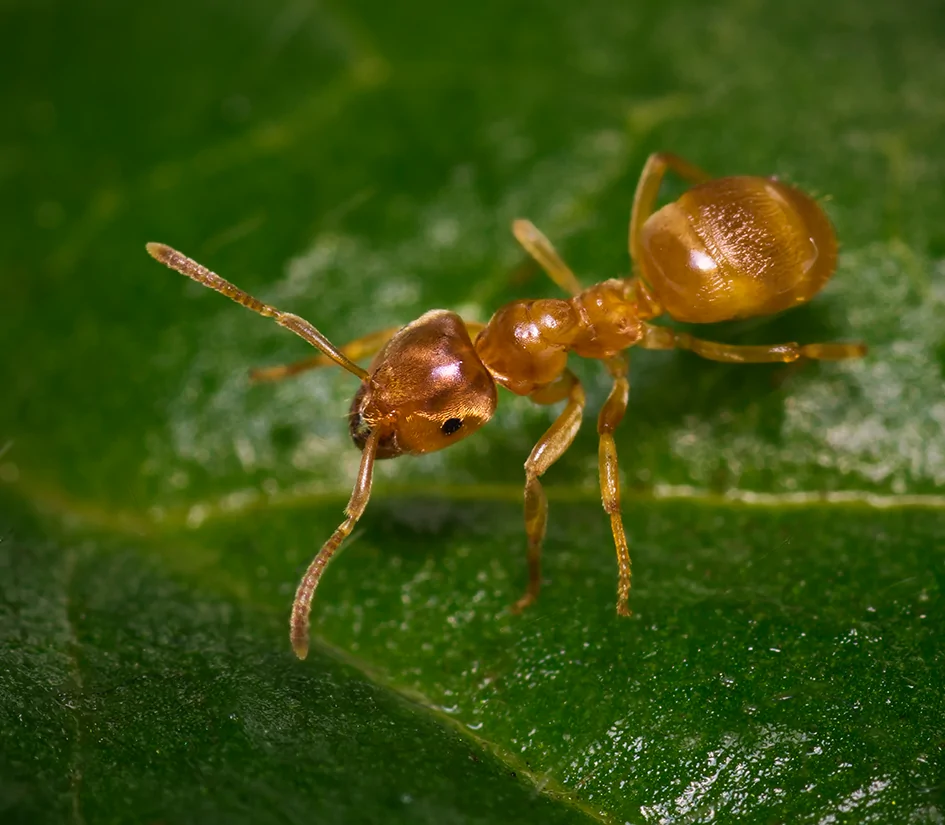
Hi-C contact map of the *Lasius flavus* genome assembly. Assembled chromosomes are shown in order of size and labelled along the axes, with a megabase scale shown below. The plot was generated using PretextSnapshot.

**Table 3.** Chromosomal pseudomolecules in both haplotypes of the genome assembly of *Lasius flavus*, iyLasFlav2.

Haplotype 1				Haplotype 2			
INSDC accession	Name	Length (Mb)	GC%	INSDC accession	Name	Length (Mb)	GC%
OZ287105.1	1	26.26	37	OZ287090.1	1	26.73	37
OZ287106.1	2	25.48	37	OZ287091.1	2	25.85	37
OZ287107.1	3	22.76	36	OZ287092.1	3	22.32	36
OZ287108.1	4	22.21	37.50	OZ287093.1	4	21.52	37.50
OZ287109.1	5	22.17	37.50	OZ287094.1	5	21.42	36.50
OZ287110.1	6	21.75	37	OZ287095.1	6	21.72	36.50
OZ287111.1	7	20.88	36.50	OZ287096.1	7	20.92	36.50
OZ287112.1	8	19.83	38	OZ287097.1	8	20.78	38
OZ287113.1	9	19.73	38	OZ287098.1	9	20.37	38
OZ287114.1	10	19.35	38	OZ287099.1	10	18.48	37.50
OZ287115.1	11	18.48	36.50	OZ287100.1	11	18.14	36.50
OZ287116.1	12	18.36	37.50	OZ287101.1	12	18.35	37.50
OZ287117.1	13	15.95	37.50	OZ287102.1	13	16.56	38
OZ287118.1	14	13.92	38	OZ287103.1	14	13.65	37.50
OZ287119.1	15	13.18	36	OZ287104.1	15	13.53	36.50

The mitochondrial genome was also assembled (length 21.36 kb, OZ287120.1). This sequence is included as a contig in the multifasta file of the genome submission and as a standalone record.

### Assembly quality metrics

For haplotype 1, the estimated QV is 57.5, and for haplotype 2, 57.0. When the two haplotypes are combined, the assembly achieves an estimated QV of 57.2. The *k*-mer completeness is 90.24% for haplotype 1, 90.77% for haplotype 2, and 99.37% for the combined haplotypes ( Figure 4).

**Figure 4.**
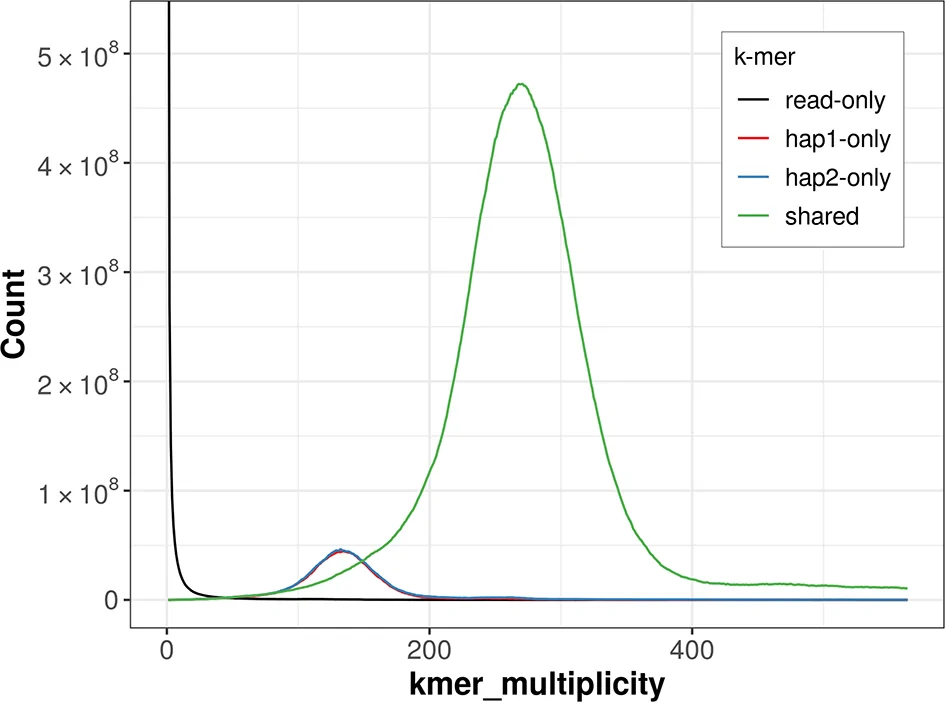
Evaluation of *k*-mer completeness using MerquryFK. This plot illustrates the recovery of *k*-mers from the original read data in the final assemblies. The horizontal axis represents *k*-mer multiplicity, and the vertical axis shows the number of *k*-mers. The black curve represents *k*-mers that appear in the reads but are not assembled. The green curve corresponds to *k*-mers shared by both haplotypes, and the red and blue curves show *k*-mers found only in one of the haplotypes.

BUSCO analysis using the hymenoptera_odb10 reference set ( *n* = 5 991) identified 97.4% of the expected gene set (single = 95.4%, duplicated = 2.0%) in haplotype 1. For haplotype 2, BUSCO v.5.8.3 analysis identified 97.9% of the expected gene set (single = 94.9%, duplicated = 3.0%). The snail plot in Figure 5 summarises the scaffold length distribution and other assembly statistics for haplotype 1. The blob plot in Figure 6 shows the distribution of scaffolds by GC proportion and coverage for haplotype 1.

**Figure 5.**
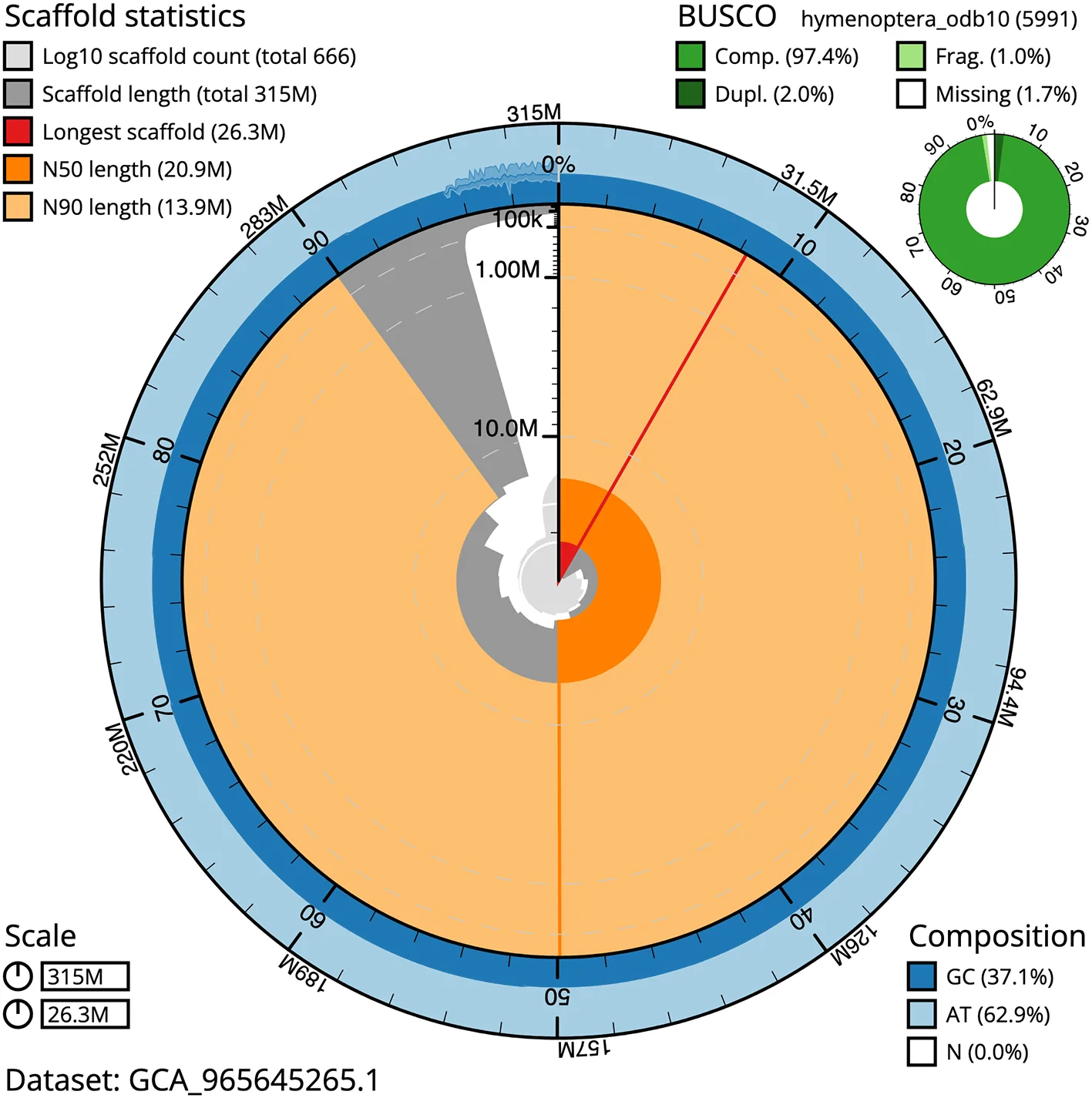
Assembly metrics for iyLasFlav2.hap1.1. The BlobToolKit snail plot provides an overview of assembly metrics and BUSCO gene completeness. The circumference represents the length of the whole genome sequence, and the main plot is divided into 1 000 bins around the circumference. The outermost blue tracks display the distribution of GC, AT, and N percentages across the bins. Scaffolds are arranged clockwise from longest to shortest and are depicted in dark grey. The longest scaffold is indicated by the red arc, and the deeper orange and pale orange arcs represent the N50 and N90 lengths. A light grey spiral at the centre shows the cumulative scaffold count on a logarithmic scale. A summary of complete, fragmented, duplicated, and missing BUSCO genes in the hymenoptera_odb10 set is presented at the top right. An interactive version of this figure can be accessed on the BlobToolKit viewer.

**Figure 6.**
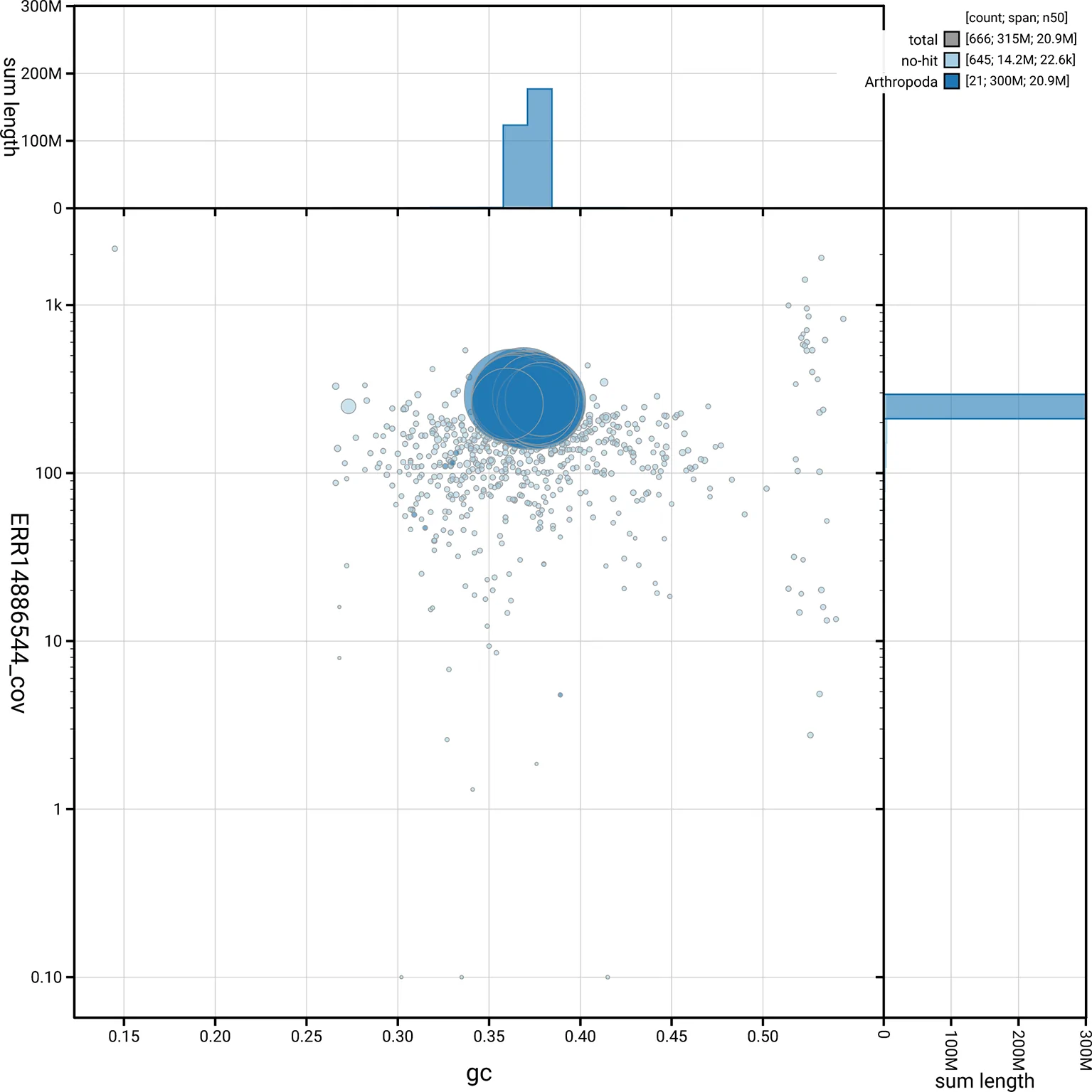
BlobToolKit blob plot for iyLasFlav2.hap1.1. The plot shows base coverage (vertical axis) and GC content (horizontal axis). The circles represent scaffolds, with the size proportional to scaffold length and the colour representing phylum membership. The histograms along the axes display the total length of sequences distributed across different levels of coverage and GC content. An interactive version of this figure is available on the BlobToolKit viewer.

Table 4 lists the assembly metric benchmarks adapted from Rhie *et al.* (2021) and the Earth BioGenome Project Report on Assembly Standards. The EBP metric, calculated for haplotype 1, is **5.C.Q57**, meeting the recommended 5.C.Q40 reference standard for samples with limited input material.

**Table 4.** Earth Biogenome Project summary metrics for the *Lasius flavus* assembly.

Measure	Value	Benchmark
EBP summary (haplotype 1)	5.C.Q57	5.C.Q40
Contig N50 length	0.81 Mb	≥ 0.1 Mb
Scaffold N50 length	20.88 Mb	= chromosome N50
Consensus quality (QV)	Haplotype 1: 57.5; haplotype 2: 57.0; combined: 57.2	≥ 40
*k*-mer completeness	Haplotype 1: 90.24%; Haplotype 2: 90.77%; combined: 99.37%	≥ 90%
BUSCO	Haplotype 1: C:97.4% [S:95.4%, D:2.0%], F:1.0%, M:1.7%, n:5 991; Haplotype 2: C:97.9% [S:94.9%, D:3.0%], F:1.0%, M:1.1%, n:5 991	S > 90%; D < 5%
Percentage of assembly assigned to chromosomes	95.46%	≥ 90%

*Notes:* The EBP summary uses log10(Contig N50); chromosome-level (C) or log10(Scaffold N50); Q (Merqury QV). BUSCO: C=complete; S=single-copy; D=duplicated; F=fragmented; M=missing; n=orthologues

**Table 5.** Software versions and sources used for *Lasius flavus.*

Software	Version	Source
BLAST	2.14.0	https://ftp.ncbi.nlm.nih.gov/blast/executables/blast+/
BlobToolKit	4.4.6	https://github.com/blobtoolkit/blobtoolkit
BUSCO	5.8.3; 6.1.0	https://gitlab.com/ezlab/busco
bwa-mem2	2.2.1	https://github.com/bwa-mem2/bwa-mem2
DIAMOND	2.1.8	https://github.com/bbuchfink/diamond
fasta_windows	0.2.4	https://github.com/tolkit/fasta_windows
FastK	1.1	https://github.com/thegenemyers/FASTK
GenomeScope2.0	2.0.1	https://github.com/tbenavi1/genomescope2.0
Gfastats	1.3.6	https://github.com/vgl-hub/gfastats
Hifiasm	0.19.8-r603	https://github.com/chhylp123/hifiasm
HiGlass	1.13.4	https://github.com/higlass/higlass
MerquryFK	1.1.0-c1	https://github.com/thegenemyers/MERQURY.FK
Minimap2	2.28-r1209	https://github.com/lh3/minimap2
MitoHiFi	3.2.2	https://github.com/marcelauliano/MitoHiFi
MultiQC	1.14; 1.17 and 1.18	https://github.com/MultiQC/MultiQC
Nextflow	24.10.4; 25.10.4	https://github.com/nextflow-io/nextflow
PretextSnapshot	0.0.4	https://github.com/sanger-tol/PretextSnapshot
PretextView	1.0.3	https://github.com/sanger-tol/PretextView
samtools	1.17; 1.2; 1.20; 1.21	https://github.com/samtools/samtools
sanger-tol/ascc	0.1.0	https://github.com/sanger-tol/ascc
sanger-tol/blobtoolkit	v0.8.0	https://github.com/sanger-tol/blobtoolkit
sanger-tol/curationpretext	1.4.2	https://github.com/sanger-tol/curationpretext
Seqtk	1.4-r122	https://github.com/lh3/seqtk
Singularity	3.9.0	https://github.com/sylabs/singularity
TreeVal	1.3.0	https://github.com/sanger-tol/treeval
YaHS	1.2.2	https://github.com/c-zhou/yahs

## Author information

Contributors are listed at the following links:

• Members of the Natural History Museum Genome Acquisition Lab
• Members of the Darwin Tree of Life Barcoding collective
• Members of the Wellcome Sanger Institute Tree of Life Management, Samples and Laboratory team
• Members of Wellcome Sanger Institute Scientific Operations – Sequencing Operations
• Members of the Wellcome Sanger Institute Tree of Life Core Informatics team
• Members of the Tree of Life Core Informatics collective
• Members of the Darwin Tree of Life Consortium

## Wellcome Sanger Institute – Legal and Governance

The materials that have contributed to this genome note have been supplied by a Darwin Tree of Life Partner. The submission of materials by a Darwin Tree of Life Partner is subject to the **‘Darwin Tree of Life Project Sampling Code of Practice’**, which can be found in full on the Darwin Tree of Life website. By agreeing with and signing up to the Sampling Code of Practice, the Darwin Tree of Life Partner agrees they will meet the legal and ethical requirements and standards set out within this document in respect of all samples acquired for, and supplied to, the Darwin Tree of Life Project. Further, the Wellcome Sanger Institute employs a process whereby due diligence is carried out proportionate to the nature of the materials themselves, and the circumstances under which they have been/are to be collected and provided for use. The purpose of this is to address and mitigate any potential legal and/or ethical implications of receipt and use of the materials as part of the research project, and to ensure that in doing so we align with best practice wherever possible. The overarching areas of consideration are:

• Ethical review of provenance and sourcing of the material
• Legality of collection, transfer and use (national and international)

Each transfer of samples is further undertaken according to a Research Collaboration Agreement or Material Transfer Agreement entered into by the Darwin Tree of Life Partner, Genome Research Limited (operating as the Wellcome Sanger Institute), and in some circumstances, other Darwin Tree of Life collaborators.

## Data Availability

European Nucleotide Archive: Lasius flavus (yellow meadow ant). Accession number PRJEB88760; https://identifiers.org/ena.embl/PRJEB88760. The genome sequence is released openly for reuse. The *Lasius flavus* genome sequencing initiative is part of the Darwin Tree of Life Project (PRJEB40665) and the Sanger Institute Tree of Life Programme (PRJEB43745). All raw sequence data and the assembly have been deposited in INSDC databases. The genome will be annotated using available RNA-Seq data and presented through the Ensembl pipeline at the European Bioinformatics Institute. Raw data and assembly accession identifiers are reported in Tables 1 and 2. Production code used in genome assembly at the WSI Tree of Life is available at https://github.com/sanger-tol . Table 5 lists software versions used in this study.
