# Supplementary material for: The genome sequence of the Yellow Meadow Ant, Lasius flavus (Fabricius, 1782) (Hymenoptera: Formicidae)
Source: Wellcome Open Res. 2026 Oct 6;11:548. Originally published 2026 Aug 26. [Version 2] doi: 10.12688/wellcomeopenres.27332.2 (PMC13658995; doi:10.12688/wellcomeopenres.27332.2)
Supplement: Peer review history

# Reviewer report: Reviewer response for version 2

**DOI:** 10.21956/wellcomeopenres.30639.r172493

**Published:** 2026-10-12

**Authors:** Jay K Goldberg

**Affiliations:** 1 John Innes Centre, Norwich, England, UK; 2 Arizona State University School of Life Sciences (Ringgold ID: 69991), Tempe, Arizona, USA

Glad to see the issue with figure 3 is fixed. That was my only major reservation.

Are sufficient details of methods and materials provided to allow replication by others?

Yes

Is the rationale for creating the dataset(s) clearly described?

Yes

Are the datasets clearly presented in a useable and accessible format?

Yes

Are the protocols appropriate and is the work technically sound?

Yes

Reviewer Expertise:

evolutionary genetics, plant-insect interactions

I confirm that I have read this submission and believe that I have an appropriate level of expertise to confirm that it is of an acceptable scientific standard.

# Reviewer report: Reviewer response for version 1

**DOI:** 10.21956/wellcomeopenres.30083.r166915

**Published:** 2026-09-17

**Authors:** Jay K Goldberg

**Affiliations:** 1 John Innes Centre, Norwich, England, UK; 2 Arizona State University School of Life Sciences (Ringgold ID: 69991), Tempe, Arizona, USA

Having now reviewed a few DToL genome reports, I can confidently say that this is one of the best and most thoroughly written ones to date! 

 As is typical of these reports, the methods and quality control assessments are clearly laid out and reported in an easy-to-interpret table. The QC analyses clearly show that the genome is of the same high-quality that I have come to expect from the DToL team. 

 What sets this report apart from others, is that the background/introduction section is more detailed. The description of the species, its taxonomic history and relationship with similar species, and various ecological relationships clearly demonstrate the usefulness of this genome to ecological/evolutionary geneticists. I look forward to seeing what the field is able to learn from it.

 My only complain, which is admittedly a minor one, is that Figure 1 is not a photo of the sequenced specimen itself. This is typically included in DToL reports and enables independent verification of ID by readers. There also appears to be a publication error as the image is also shown as Figure 3, instead of the Hi-C contact map. Despite the otherwise high-quality of this report, Figure 3 will need to be corrected before I can approve it without any reservations.

Are sufficient details of methods and materials provided to allow replication by others?

Yes

Is the rationale for creating the dataset(s) clearly described?

Yes

Are the datasets clearly presented in a useable and accessible format?

Yes

Are the protocols appropriate and is the work technically sound?

Yes

Reviewer Expertise:

evolutionary genetics, plant-insect interactions

I confirm that I have read this submission and believe that I have an appropriate level of expertise to confirm that it is of an acceptable scientific standard, however I have significant reservations, as outlined above.

# Reviewer report: Reviewer response for version 1

**DOI:** 10.21956/wellcomeopenres.30083.r166913

**Published:** 2026-09-09

**Authors:** Jurate De Prins

**Affiliations:** 1 Belgian Institute of Natural Sciences, Brussels, Belgium; 2 Flemish Entomological Society, Vrieselhof, Belgium

I am a lepidopterist by specialisation, but also an editor-in-chief of the Flemish Entomological Society, dealing with all insects. Please excuse me if I am not going into detail, but review from a general point of view.  There are very positive points in this article: it is very clearly explained how the studied specimen was identified; moreover, the identification is based not only on DNA barcode but, as the author writes, "can be identified relatively easily by the uniformly yellow colour of the workers and long setae on the metasoma, amongst other features." It is important that this species is a "familiar sight in European grassland", and can be identified without complicated procedures of sampling and sending it to Canada. The other very positive point is that it is mentioned in this article that Lasius flavus forms a part of complex food chains like providing areas to lay eggs to other insect orders or serving as food for birds. Since this is important information deals with different insect orders, it would be advisable to write the order name in Latin for all insects dealt with in this article ants-Hymenoptera, eggs of Orthoptera, aphid eggs Hemiptera. 

 Regarding the molecular protocol I see that still fresh  samples were shipped on dry ice to the Wellcome Sanger Institute. My very kind wish and recommendation is that our molecular experts could work with earlier collected and collection samples. It is already possible in Australia; please see my monograph, so it should be possible in the leading European molecular institutions as well. Gavin Broad is a devoted collection curator-in-charge at the NHM, so it will open a new area of research if whole genomes of the collection material could be studied.  

Are sufficient details of methods and materials provided to allow replication by others?

Yes

Is the rationale for creating the dataset(s) clearly described?

Yes

Are the datasets clearly presented in a useable and accessible format?

Yes

Are the protocols appropriate and is the work technically sound?

Yes

Reviewer Expertise:

Taxonomy, Bioinformatics, Karyology

I confirm that I have read this submission and believe that I have an appropriate level of expertise to confirm that it is of an acceptable scientific standard.

## References

[1] : Diversity of Australian Ornixolinae (Lepidoptera: Gracillariidae) with taxonomic and nomenclatural acts within the related taxa (Acrocercopinae and Gracillariinae) based on the evidence of museomics, bionomics, and mitogenomics. *Zootaxa* .2025;5616(1) : 10.11646/zootaxa.5616.1.11-340 10.11646/zootaxa.5616.1.1 40173480
